# Editorial: Applied Microbiology for Chemical Syntheses

**DOI:** 10.3389/fmicb.2017.01931

**Published:** 2017-10-11

**Authors:** Robert Kourist, Javier González-Sabín, Bettina Siebers, Mattijs Julsing

**Affiliations:** ^1^Lehrstuhl für Bioprozesstechnik, Technische Universität Dortmund, Dortmund, Germany; ^2^Department of Technichal Chemistry, Process Engineering and Biotechnology, Institute of Molecular Biotechnology, Graz University of Technology, Graz, Austria; ^3^EntreChem SL, Edificio Científico Tecnológico, Oviedo, Spain; ^4^Molecular Enzyme Technology and Biochemistry, Biofilm Centre, Centre for Water and Environmental Research, University of Duisburg-Essen, Essen, Germany

**Keywords:** biotechnology, biocatalysis, protein engineering, enzyme cascade reaction, metabolic engineering

The last years have seen a tremendous progress in the tools and techniques in microbiology. Anyone who has ever conducted a sanger-sequencing him- or herself will appreciate that this is now an automated standard-technique; isolation of restriction enzymes is a tale of a long forgotten past; the synthesis of the gene was in the early 2000s a challenging project and was in the late 2000s a considerable investment. Now, the cost is negligible. Similarly, the great progress in systems biology is producing data at an exponentially increasing rate. A few years ago, metabolic engineering meant a major analytic effort. Now, modern—omics technologies generate a systematic and quantitative understanding of the cells. For instance, quantitative data on metabolite concentration and molecular flux can be correlated with knowledge on regulation obtained by transcriptomics and proteomics. This makes it possible to identify metabolic bottlenecks and develop strategies for the knowledge-based engineering of cells.

While the list of these achievements could be extended much more, these examples clearly illustrate how much faster biotechnological research has become. The tremendous progress has led to coining of the term “synthetic biology,” which among other meanings, expresses the trend to a quantitative understanding of biology and hence, the possibility to apply engineering concepts and modular thinking to biological questions. An interesting question, however, is to which extent does this progress translate to a viable solution of modern society's problems? So far, biotechnology has undoubtedly made several very important contributions. The recombinant production of therapeutic proteins, such as insulin, reduce the risk of contamination by infectious agents, and allow the design of non-natural variants of these proteins with better therapeutic properties. Modern antibody technologies have an already noticeable, but still not exploited potential for therapy. In chemical synthesis, enzymes have emerged as often-used and reliable method for the synthesis of fine chemicals, pharmaceutical ingredients, and bio-based products (Gómez-Baraibar et al., 2016). However, whole-cell biocatalysts and enzymes always have to be compared to alternative chemical methods. Domínguez de Maria and Hollmann point out in an interesting perspective article that an analysis of cost and sustainability of a process must consider the entire process, and that biotechnological processes are not always the best. Nevertheless, they show that several approaches, including the use of non-conventional media and solvent free reactions, so-called “neat” conditions, have a still unexploited synthetic potential. A wide implementation of biotechnological processes requires cost-efficient reactions that offer clear advantages over the state-of-the-art, either in terms of efficiency or of a superior quality. The present research topic gives an overview how Microbiology can provide the catalysts for the development of sustainable processes, and how it offers tools for the optimization of the biocatalysts. Several articles cover the expansion of the available sequence space by targeted enzyme discovery from extremophilic organisms and by enzyme engineering. Several novel and emerging reactions such as lignin-degrading β-etherases are presented. The topic then summarizes the interaction of enzymes in whole-cell biocatalysts and artificial enzyme cascades and how they can be optimized. Finally, the topic presents the utilization of nature's catalytic diversity for the synthesis of complex natural products.

Extremophilic organisms offer a still untapped diversity of biocatalysts with unusual properties for biotechnological applications. Zeldes et al. give a comprehensive review on recent trends and developments of the approach. They highlight the availability of genetic tools in several (hyper)thermophilic Bacteria and Archaea, such as *Caldicellulosiruptor, Sulfolobus, Thermotoga, Thermococcus*, and *Pyrococcus*, and the current use of this organisms as new platform organisms. Santiago et al. introduce to potential industrial applications of cold-active enzymes, and the molecular basis for the high activity at low temperatures. While thermostable enzymes are already used in industry, the molecular mechanism of their high stability and the principles determining their catalytic properties are still not fully understood. A variety of strategies for thermoadaptation has been reported that are employed by different enzymes. Sayer et al. resolved the structure of a new thermostable esterase, and rationalize the substrate recognition by an analysis of the active-site pocket and a “minimal” cap domain. In this line, Khan et al. present the entrance region of alpha/beta-fold hydrolases as a key structural element in this important class of enzymes. Two reports show the hitherto mostly overlooked ability of microorganisms from extreme habitats to degrade polysaccharides. The use of these organisms makes it possible to utilize polysaccharides such as cellulose, chitan, and cellulose at high salinity or elevated temperatures. Sorokin et al. describe several new archaeal strains isolated from hypersaline lakes that are able to utilize cellulose and chitin for growth. Several of the isolated represent novel phylogenetic lineages within the class *Halobacteria*. Gavrilov et al. report the isolation of a hyperthermophilic archaeon with the unusual ability to degrade xylan. Genome sequencing revealed the presence of a novel glycosidase with a unique five-domain structure. The work from Gavrilov et al. underlines that the classical combination of enrichment, comparative genomics, and biochemical approach is an efficient approach for the targeted identification of new biocatalysts from extremophiles. On the one hand, frequent difficulties to express enzymes from extremophiles in mesophilic hosts make functional metagenomics difficult. On the other hand, the unusual properties of the biocatalysts are associated with a very low degree of similarity to known biocatalysts, which makes sequence-function predictions very challenging. In this line, Masuch et al. developed a very interesting combined bioinformatics and functional metagenomics approach for the targeted identification of enzymes with little similarity to known proteins. Using this method, they were able to identify a novel lipase with little similarity to known biocatalysts. Riedel et al. approach the analysis of structural differences between enzymes from mesophilic and extremophilic organisms from a different angle. They report the functional characterization of bacterial ene-reductase that has high similarity to thermostable esterases. By introduction of a salt-bridge, they were able to increase the half-life time at moderate temperatures more than two-fold.

While the enzyme isolation of enzymes from extremophiles gains momentum, the well-known mesophilic bacteria still harbor a vast diversity of biocatalysts with interesting synthetic properties. While Bacteria and Archaea are efficient in the depolymerization of natural polysaccharides, lignin-degradation has been mostly attributed to fungi. Picart et al. present a highly interesting class of β-etherases from α-proteobacteria that cleave the predominant bond in lignin and play a potentially important role in biorefinery. Nowak et al. report an NADH oxidase from *Lactobacillus pentosus* that produces water instead of the detrimental hydrogen peroxide that is formed by most NADH oxidases. They demonstrate the usefulness of the enzyme in the regeneration of oxidized nicotinamide cofactors. Maimanakos et al. report the discovery of several enantioselective decarboxylases. Using a combined comparative genomics and functional screening approach, they were able to identify several novel arylmalonate decarboxylases. This enzyme is quite rare and its natural natural function remains unknown (Miyamoto and Kourist, 2016). Similar to the work from Gavrilov et al. the approach underlines of the combination of genome sequencing and functional approaches.

Besides the targeted identification of new biocatalysts, enzyme engineering and reaction engineering are highly efficient for an expansion of the catalytic scope of biocatalysis. Fink et al. report an iron-dependent oxidase with the rare ability to convert alkenes to ketones. By targeted saturation mutagenesis of amino acids in the active site, they were able to achieve a significant improvement of the activity and an expansion of the substrate scope.

While enzyme engineering is the method of choice for the optimization of enzyme selectivity, substrate spectrum and, to some extent, specific activity, increasing the operational stability can be both obtained by enzyme and reaction engineering. Aßmann et al. demonstrate the efficiency of enzyme immobilization for the increase of the biocatalytic productivity with the example of an enantioselective decarboxylase. Agarwal et al. demonstrate also bio-based materials are efficient carriers for immobilization purposes.

Besides the discovery and engineering of individual biocatalysts, the fine tuning of intracellular enzyme cascade reactions is a dynamic and rapidly developing research field. Blank et al. present a new process for the production of anthranilate in *Pseudomonas putida*. Deletion of an operon encoding an anthranilate phosphoribosyltransferase and an indole-3-glycerol phosphate synthase reached a strain that achieved, under fed-batch conditions, the production of anthanilate with an impressive volumetric yield of 1.5 g L^−1^. Also with *P. putida*, Lindmeyer et al. demonstrate a phenomenon that is actually decisive for cell engineering and fermentation, but is still poorly understood. They analyzed the variability in subpopulation formation in an engineered strain bearing recombinant styrene monooxygenase. Analysis of the expression of a GFP-fusion with a cell-sorter revealed at least two distinct subpopulations. Plasmid copy numbers could be ruled out as source, which made regulatory phenomena the most likely explanation. The results shed light on the phenotypic heterogeneity of recombinant bacteria, and of the risk of relying on parameters that represent the average of different subpopulations during cultivation which might complement each other, leading to deceiving data. Markošová et al. demonstrate the scalability of *P. pastoris* in the production of recombinant proteins. Taniguchi and Wendisch investigated the regulation of production strains. They overexpressed putative sigma factors under the control of an IPTG-inducible promoter. Overexpression of the sigma factor H increased the expression of riboflavin biosynthesis, pentose pathway, and FMN and NADPH-dependent enzymes.

While the above works underline the methodological process of enzyme catalysis, the synthesis of complex natural products is an example *per excellence* how biotechnology can contribute to the synthesis of bioactive products for the pharmaceutical and chemical industry. Nature offers an endless pool of bizarre and sophisticated molecular entities with desirable drug-like properties, rendering them ideal starting points for development of pharmaceuticals (Mishra and Tiwari, 2011). Actually, the chemical structures of natural products (NPs) are the result of an on-going combinatorial chemistry performed by living organisms over millions of years, providing them multiple advantages related to their growth and survival. This explains the unique ability of these entities to specifically interact with biological target molecules. Thus, the history of NPs in drug discovery has been extraordinarily successful over the past century, highlighted by prominent examples such as the antitumoral agents taxol, vinblastine or doxorubicin, the immunosuppressants cyclosporine, and rapamycin, or the cholesterol-lowering agents statins, the top-selling drugs today. Anyway, novel compounds are still needed to address uncovered medical needs, like infectious diseases, or cancer. In this context, genome mining, which is focused on searching a genome for genes that encode enzymes involved in a particular process, has driven the discovery of novel NPs. In the contribution of Ye et al. genome mining of the mithramycin producer *Streptomyces argillaceus* ATTC 12956 revealed 31 gene clusters for the biosynthesis of secondary metabolites. Further identification of encoded compounds showed six new alkaloids named argymicins P with antibiotic activity.

On the other hand, the recent advances in molecular biology have made biocatalysis a widely-used tool in many synthetic reaction sequences in both academia and chemical industry. Actually, the technique of engineering natural products biosynthetic pathways, redefined as combinatorial biosynthesis (Zhou et al., 2008) now allows the isolation, identification and overexpression of any naturally occurring enzyme in a specific carrier and the use of the recombinant enzyme for a particular transformation. These “biosynthetic enzymes” have already been used as biocatalysts in macrocyclization, glycosylation, and acylation reactions. Now, the next step to afford a new dimension to biocatalysis consist in expanding the application of the enzymatic toolbox to the modification of complex molecular scaffolds common to many pharmaceutical targets isolated from nature, leading to new drugs with remarkable improved activity, stability, and pharmacokinetic properties. Traditionally, some recalcitrant issues associated to NPs were the chemical fragility, structural complexity, and functional diversity which make the transformation of a specific functional group a truly challenging task. Enzymes can circumvent most of the aforementioned problems seeing as they exhibit high selectivity and operate under mild conditions in both aqueous and organic media. As a result, a biocatalytic approach could hypothetically reduce the number of protection/deprotection steps and introduce structural diversity inaccessible via conventional chemical synthesis. This is particularly relevant seeing as only 6% of the 1,024 new molecules drugs approved for the treatment of human diseases in the past three decades (1981–2008) are directly isolated NPs. In contrast, over half of these molecules (57%) are either NP derivatives obtained by semi-synthetic approaches or formally synthetic compounds that are structurally related to them. In this regard, some of contributions included in this Research Topic are focused on this issue. For example, the glycosyltransferases (GTs), discussed independently by Pandey and Sohng and Schmid et al. are an attractive class of enzymes for post-modifications of NPs by conjugating diverse types of sugar appendages. Actually, NP glycosides are widespread in nature and have a deep impact on human daily life. More interestingly, microbial originated glycosylated molecules such as doxorubicin, staurosporine, or vancomycin contain highly modified deoxysugars, which have shown to play a crucial role in executing the biological functions. In their perspective article, discuss the challenges and opportunities of the bacterial GTs, focusing on the classification, screening, and engineering strategies to alter the substrate specificity. On the other hand, the contribution of Görner et al. reports a combination of biotechnological, chemo-enzymatic, and chemical reaction cascades for the efficient generation of novel bioactive analogs from the dolabellanes NPs family. Thus, the employment of a CotB2 diterpene synthase mutant (W288G) led, instead of the native product cyclooctat-9-en-7-ol, to (1R,3E,7E,11S,12S)-3,7,18-dolabellatriene. Then, the bioactivity of this olefinic macrocycle was diversified by the action of combined chemical and enzymatic methods. Similarly, although not formally complex natural products, the multi-enzymatic cascade reaction described by Enoki et al.enabled the synthesis of diastereomerically pure γ-oxyfunctionalized α-amino acids from inexpensive reagents. Specifically, three different enzymes, namely a *N*-acylamino acid racemase, a L-selective aminoacylase, and a stereoselective isoleucine dioxygenase were coupled in a stepwise fashion to produce L-methionine-(*S*)-sulfoxide.

In conclusion, the topic provides an overview on current trends and shows the substantial contribution of Microbiology on the way to a clean, sustainable chemical, and pharmaceutical industries. The tremendous methodological progress in the field will expand the synthetic scope of Natural Catalysts even more in the near future.

## Author contributions

RK devised and wrote the editorial. JG and MJ contributed to the text. BS revised the manuscript and edited the part about Archaeal Enzymes.

### Conflict of interest statement

The authors declare that the research was conducted in the absence of any commercial or financial relationships that could be construed as a potential conflict of interest.
